# China's Particle Therapy Equipment Market: Opportunities Outweigh Challenges

**DOI:** 10.3389/fpubh.2020.602776

**Published:** 2020-12-01

**Authors:** Zhongying Dai, Yuanyuan Ma, Qiang Li

**Affiliations:** Department of Radiation Medical Physics, Institute of Modern Physics, Chinese Academy of Sciences, Lanzhou, China

**Keywords:** particle therapy, Chinese particle therapy equipment, medical device market, medical device industry innovation, medical device regulation (MDR)

## Abstract

Since 2019, China has been the second largest medical device market in the world. At present, high-end radiotherapy equipment such as particle therapy system has a huge market potential due to the grim situation of cancer prevention and control and the growth of people's wealth in China. However, China's MedTech industry, especially the particle therapy equipment field, still faces the influence of policy, fluctuation of market demand, strengthening of industry supervision, and even geopolitical realities. This paper reviews the market prospect of particle therapy medical devices from the perspective of China's medical device policy and demand information analysis, which is conducive to the research on the industrial layout of particle therapy medical physics, and also helps high-performance medical device manufacturers to expand their business visions. MedTech manufacturers should actively adjust their business strategy and implement scientific and technological innovation on the basis of compliance with industry regulatory requirements in order to seize opportunities from challenges and gain profits growth.

## Introduction

The Chinese medical device market is fostered by the burgeoning demand from the increasingly affluent and a rapidly aging population and the rising prevalence of chronic diseases, such as diabetes, heart disease, and cancer.

Today, the market for medical devices in China is rapidly developing, far exceeding the country's economic growth rate. Currently it is increasing in value by around 20% a year, and it was worth an estimated value of $96.3 billion in 2019, making it the second largest medical device market in the world after the United States^1^. Among them, China's radiotherapy equipment market increased from 5.83 billion yuan in 2008 to 26.9 billion yuan in 2015, with a compound annual growth rate of 24.42% (1, 2).

Particle therapy equipment in particular is in great need in China.

From 2015 to 2020, a total of 77 proton and heavy ion center projects have been reported in China, including two heavy ion (Wuwei and Shanghai) and four proton therapy projects in operation, 26 proton projects under construction, and 60 proposed items. Many hospitals in China have signed purchase agreements for proton heavy ion radiotherapy equipment. Enthusiasm for investment presents the potentially attractive and capacious market future of particle therapy equipment.

However, profit growth in China's MedTech industry still faces the influence of policy, shifting of market demand, strengthening of industry supervision, and even geopolitics. Specifically, the shifting of domestic demand, regional share, and strict supervision are the challenges faced by the high-end medical equipment industry, especially the particle therapy equipment field.

## Seeking Opportunities from Challenges

### Challenges: Localization of Market Demand

China's “made in China 2025” initiative calls on China's top hospitals to increase the use of domestically produced equipment by 50% by 2020, focusing on the development of high-performance equipment, such as diagnostic imaging equipment, robotic surgical instruments, etc. (3). The trend of medical device market demand is obviously affected by the policies and incline to be localized.

The state pushes forward the implementation of demand localization strategy through centralized bidding policy and governmental procurement redirection. Those technology roadmap and business strategy of medical device products with access to the Chinese market need to adapt to the shifting demand (4).

The state-owned public and private hospitals respond quickly for the localization strategy in China. In 2019, six quotas of proton therapy equipment were obtained by large-scale regional public hospitals, at least half of which are to be locally made. Although Chinese medical device manufacturers are the leaders in the domestic market, hospitals and healthcare providers nationwide prefer imported devices, especially when purchasing innovative and high-tech devices. Significant scale of demand for foreign-made, high-end devices is also fueled by continuous growth of China's aging population, and driving forces to improve the quality of care by the Chinese government. Obviously, the strategy of using legal commercial means to transform foreign brand products into local-made ones helps to cope with the changes in market demand trend.

### Challenges: Regionalization of Market Share

Due to the worry that hospitals introduce large-scale medical equipment blindly, resulting in waste of resources, the state has strengthened the market-oriented demand management of this equipment (5). With regard to large radiotherapy equipment such as particle therapy system, linear accelerator, and PET, the state has planned the allocation area according to the actual situation of the coverage capacity of medical service and the diagnosis and treatment level of medical institutions, especially the carbon ion proton treatment system. In 2018, the authority of the China medical device in central government came up with a medical equipment quota management policy, in which the basic market share of proton therapy systems in China was allocated macroscopically, with at least 10 proton therapy systems to be disposed by the end of 2020 (6).

For those medical equipment companies that really want to maximize profits by utilizing market demand, it is time to adjust their product, supply chain, and commercial settings flexibly. In this way, medical equipment manufacturers have the chance to seek out high profits in the value chain by providing unique high-quality equipment that meets both the market demand and market regulatory requirements.

### Challenges: Internationalization of Market Supervision

The concept of China's medical device regulation also helps to standardize the medical device market. In recent years, the supervision notion has gradually integrated with the international mainstream regulatory mechanism, laying stress on ensuring the safety and effectiveness of public use of medical devices through strengthening the awareness of subject responsibility (7, 8). Since 2014, China has implemented the policy of “registration before production license” and paid more attention to the supervision of medical devices after marketing (9). The system of medical device marketing license holder (MAH) defines the responsibilities of product marketing holders from the perspective of “responsibility”; China's “Internet +” plan of action has sped up the realization of information traceability of medical device products, through the construction and implementation of medical device market license information supervision and device unique identification systems (10). To seize market opportunities and make huge profits, the enterprises who develop particle therapy system with complex components should focus on the following aspects: internally, improving information management level and product quality control system, cutting down on time and energy wasting; externally, strengthening industry self-discipline, complying with regulatory requirements spontaneously and saving cost of market access, capturing new development opportunities, and seizing market opportunities.

The market supervision principle of Chinese government requires that MedTech manufacturers must comply with obscure requirements. In view of this industry and market trend, the medical device industry tends to adopt a localization strategy, which helps to win purchase orders on the one hand and has the additional benefits of reducing costs and enhancing market familiarity on the other hand. The foreign medical device enterprise, which occupy the high position of the value chain, also needs to be adjusted to maintain brand advantage and earn high profits. MedTech companies from the developed world should find other ways to enter China such as finding OEM partners, licensing technology, establishing factories, etc.

### Opportunity: Medical Device Industry Innovation Encouraged

Despite many challenges, both foreign and domestic medical device manufacturers still have many opportunities to make profits under the innovation strategy. In order to achieve its goal of promoting China's economy up the value chain, creating “national champions” and lowering dependence on foreign imports, China has made the development of the country's biomedical and high-end medical device manufacturing sector a key priority (11, 12). Incentive policies such as the special plans of medical equipment and technical innovation have been formulated. The medical device industry is required to make breakthroughs in frontier and common technology, especially large-scale medical devices with intensive high-tech applications, wide interdisciplinary cross-sections, and significant technology integration, such as particle therapy systems, nuclear magnetic resonance instrument, CT, and 10 other categories of advanced medical equipment.

MedTech innovation was encouraged with a series of policies intensively promulgated throughout the development process of China's medical equipment industry. In 2013, the National Health Commission of China launched the application development and promotion plan of domestic large-scale equipment; in 2015, China grouped medical devices into the key realm of the medical industry, and was determined to accelerate the localization of medical device manufacturing during the 13th Five Year Plan period (13).

The government's “Made in China 2025” initiative for improving industry efficiency, product quality, and brand reputation will further boost the development of domestic medical device manufacturing. The revision of NMPA (the National Medical Products Administration) regulations has listed medical device innovation as a priority, which speeds up the market access of innovative products. By the end of 2019, NMPA has approved the market access of 73 innovative medical device products, including the carbon ion therapy system independently developed by China (14).

In addition, domestic medical device manufacturers are encouraged to increase investment in technology and technological innovation to enhance long-term competitiveness and move up the value chain. Some local manufacturers are growing stronger and more competitive with foreign suppliers, such as Lanzhou Kejin Taiji, Shanghai Lianying, Beijing Guoke ion, Shenzhen Aowo, Xinhua medical and Neusoft Medical Co etc., which have been competitive in CT, gamma knife, linac, carbon ion therapy system, and other products in recent years.

## Discussion

At present, China's medical device market is full of uncertainty. In addition to being associated with the outbreak of a new coronavirus disease (officially known as COVID-19), the medical device industry market is expected to be alert to the impact and consequences of the trade war between the United States and China.

However, opportunities outweigh challenges in China's high-end medical device market nowadays accordingly.

Specifically, the influence of policy, fluctuation of market demanding, industry supervision enhancement, and even geopolitical realities are the challenges faced by the high-end medical equipment industry, especially the particle therapy equipment field. The opportunity comes from the flexible adaptation of policy and market demand fluctuation through technological innovation and product improvement.

In the next national planning cycle, China's 14th Five year plan (2021–2025), the national intellectual property system will be further strengthened, and the more mature supervision mode of the medical technology market, such as two vote system and GMP (Good Manufacturing Practice) / GSP (Good Supply Practice) management evaluation system, will be improved. With the rapid development of a highly digital medical device ecosystem, the market access path of innovative products is expected to be shortened.

In view of this, the opportunities for localized innovative medical devices are greater, compared with the fierce market competitive pattern of China's medical device market, especially in high-end equipment.

## Author Contributions

ZD: data collection and drafting papers. YM: make important revisions to the paper. QL: approval of final papers to be published. All authors contributed to the article and approved the submitted version.

## Conflict of Interest

The authors declare that the research was conducted in the absence of any commercial or financial relationships that could be construed as a potential conflict of interest.

## Footnotes

^1^Available online at: https://ibc-static.broad.msu.edu/sites/globaledge/medc/industry-mpi/pdfs/medical-devices-MPI-Insights-and-Rankings-2017.pdf
